# Changes in screening, diagnosis, management, and outcomes of gestational diabetes during the COVID-19 pandemic: A systematic review

**DOI:** 10.1016/j.heliyon.2024.e31943

**Published:** 2024-05-25

**Authors:** Kowsar Qaderi, Ahmadreza Shamsabadi, Arezoo Haseli, Sajjad Ghane Ezabadi, Leila Asadi, Younes Jesmani, Mehri Kalhor, Bita Jamali, Mehrnaz Kajbafvala, Rasa khodavirdilou, Aida Mohammadi, Dara Rasoal

**Affiliations:** a Clinical Research Development Center, Motazedi Hospital, Kermanshah University of Medical Sciences, Kermanshah, Iran; b Department of Health Information Technology, Esfarayen Faculty of Medical Science, Esfarayen, Iran; c Multiple Sclerosis Research Center, Neuroscience Institute, Tehran University of Medical Sciences, Iran; d School of Nursing and Midwifery, Tehran University of Medical Sciences, Tehran, Iran; e Students Research Committee, Kermanshah University of Medical Sciences, Kermanshah, Iran; f Department of Midwifery and Reproductive Health, School of Nursing and Midwifery, Shahid Beheshti University of Medical Sciences, Tehran, Iran; g Department of Nursing and Midwifery, Comprehensive Health Research Center, Babol Branch, Islamic Azad University, Babol, Iran; h Rehabilitation Research Center, Department of Physiotherapy, School of Rehabilitation Sciences, Iran University of Medical Sciences, Tehran, Iran; i Department of Reproductive Biology, Faculty of Advanced Medical Sciences, Tabriz University of Medical Sciences, Tabriz, Iran; j School of Health and Welfare, Dalarna University, Falun, Sweden

## Abstract

**Background:**

Gestational diabetes mellitus (GDM) is the most common medical complication of pregnancy, and it can lead to complications for the mother and the infant/fetus. This was especially evident during the COVID-19 pandemic. Therefore, the present systematic review aimed to describe the changes in screening, diagnosis, management, and outcomes of gestational diabetes during the COVID-19 pandemic.

**Methods:**

The systematic review was conducted from December 2019 until January 1, 2022. To find articles related to the purpose of the study, PubMed, Scopus, Web of Science, and WHO were searched using relevant and validated keywords using MeSH/Emtree.

**Results:**

In total, 675 entries were ascertained from the database inquiry, and 17 scholarly works were deemed suitable for inclusion in the final review. The salient conclusions derived from this review were as follows: (a) During the COVID-19 pandemic, there was a significant decrease in the use of OGTTs and a rise in the use of HbA1c assays for both GDM screening and diagnosing. (b) A predominant number of physicians incorporated some variation of telemedicine to remotely supervise and conduct follow-up evaluations of patients with GDM. Various strategies are presented for the provision of prenatal care to women afflicted with GDM, such as concentrating on high-risk demographics, the initiation of lifestyle modifications at early stages, and the implementation of remote patient monitoring techniques. The 'single test procedure' is identified as the most suitable for the preliminary screening of GDM. The OGTT should be assigned clinical precedence in patients at high risk during the ongoing COVID-19 pandemic. Additionally, Medical Nutrition Therapy (MNT) was established as the primary management strategy, and the most influential determinant of the transition from dietary adjustments to pharmacotherapy was the Fasting Blood Glucose (FBG) levels during the second trimester.

**Conclusion:**

Suggested strategies for GDM screening and management during the pandemic integrated into routine antenatal care, emphasized the importance of remote diabetes education and technology utilization during health emergencies.

## 1 Introduction

Gestational Diabetes Mellitus (GDM) is characterized as hyperglycemia with onset or first recognition during the second or third trimester of pregnancy that was not overt diabetes before to gestation [1]. According to The International Association of Diabetes and Pregnancy Study Groups (IADPSG) Consensus Panel, initial recommendations are that fasting plasma glucose (FPG) of ≥5.1 mmol/L should be classified as GDM in early pregnancy [2]. The IADPSG criteria employ a one-step method using a 75-g 2-h oral glucose tolerance test (OGTT), where a single abnormal value is sufficient for a GDM diagnosis [1,[3], [4], [5]].

This condition typically emerges around the 24th to 28th week of gestation and if not managed appropriately, it can lead to various health complications for both mother and baby [1] including neonatal hypoglycemia, macrosomia, preeclampsia, preterm delivery, and polyhydramnios [6]. The International Diabetes Federation has reported its prevalence as being more than 14.0 % in 2017 and this included approximately 21.3 million live births [[7], [8], [9]]. Changes in the blood glucose of pregnant women with gestational diabetes should be reported immediately to a healthcare professional [6].

COVID-19 posed significant challenges to healthcare systems all around the world. Many pregnant women, were infected with the COVID-19 virus [10]. It seems that physiological changes during pregnancy may potentially lead to more infectious diseases during pregnancy. In previous epidemics, such as the type 1 influenza epidemic, pregnant women were more susceptible to severe illness and mortality than the general population [11]. There are increased risks of hospitalization (5.4 times), mechanical ventilation (1.7 times), and intensive care unit (ICU) admission (1.5 times), for pregnant women with confirmed COVID-19 compared with non-pregnant women. There is twice the risk for complications such as diabetes and preeclampsia in pregnant women with COVID-19. The GDM prevalence in women with COVID-19 is approximately 7.5–11.6 % [5].

Nevertheless, amid the COVID-19 pandemic, curbing viral transmission through physical distancing emerged as a crucial public health measure. Amid the imperatives of social isolation and mitigating COVID-19 transmission risks, patients must minimize hospital visits, avoid spending long periods at the hospital, and limit face-to-face contact with healthcare practitioners. The 75g 2-h Oral Glucose Tolerance Test (OGTT) was among the recommended diagnostic measures under provisional international guidelines, which aimed to minimize potential risks to pregnant women [12]. With the changes in screening of women who had been diagnosed with GDM, significant outcomes could have been influenced. The study by Kasuga et al. (2020), it was stated that the Japanese Society of Diabetes and Pregnancy published the Japanese GDM diagnostic strategy in the evolving COVID-19 pandemic, on the April 10, 2020. Applying this strategy, which was a modification of the United Kingdom and Australian guidelines, 41 % of patients with GDM who should be treated, might not be diagnosed when the Japanese GDM diagnostic strategy during the COVID-19 pandemic is used [13].

In varied contexts, the percentage of women diagnosed with GDM solely based on fasting blood glucose (FBG) ranged between 26 % in Hong Kong and 74 % in Barbados. It's important to note, however, that using FBG as the only diagnostic criterion would result in a significant underdiagnosis, with 40–50 % of women with GDM potentially remaining undetected. This calls for a reconsideration of diagnostic criteria and procedures, particularly under conditions of social distancing and limited medical interactions [12]. Although the results of the studies indicated that telemedicine interventions can be beneficial in relation to HbA1c and fasting blood sugar, more research should be undertaken in this area [14].

Anecdotal evidence suggests that the control of GDM may have been compromised during periods of pandemic-induced lockdowns. Essential elements of managing diabetes encompass dietary guidance, self-monitoring of blood glucose, and, when required, regular administration of insulin. Instantaneous adjustments in treatment based on glycemic indices are crucial. Such a comprehensive strategy inherently requires frequent outpatient consultations [15]. The management of GDM during the COVID-19 pandemic seems to have been challenging and unsatisfactory [16].

While several studies have scrutinized the direct impacts of COVID-19 on pregnant individuals, the pandemic's indirect repercussions on pregnancy outcomes remain to be thoroughly explored. A critical concern in pregnancies complicated by GDM is the mitigation of adverse maternal and neonatal outcomes, predominantly achieved through adequate glycemic control [15]. Some systematic reviews have recently been conducted on maternal and perinatal outcomes during the COVID-19 pandemic. The findings of Chmielewski et al. study [17] showed that although most maternal outcomes such as perinatal deaths, stillbirth, ectopic pregnancies, and maternal distress deteriorated during the COVID-19 pandemic, there were no remarkable effects on other outcomes such as maternal gestational diabetes [17]. In 2020, Miola et al. (2020) asserted that differences between studies concerning complications pertaining to COVID-19 in women with GDM exist. Findings of four studies relating to pregnant woman with GDM who have had COVID-19 showed that there was limited evidence that COVID-19 exacerbated the problem [18]. Wei et al., in 2021 in a systematic review and meta-analysis, concluded that severe COVID-19 was vigorously associated with gestational diabetes [19]. In 2021, Eberle et al. (2021). Found that the concurrence of GDM and COVID-19 can lead to adverse outcomes for mother and child [14].

As of now, we lack comprehensive data regarding the changes of screening, diagnosis, management, and outcomes of GDM, amidst the COVID-19 pandemic.

## 2 Materials and methods

### 2.1 Study design

A systematic search strategy was employed across various databases, including PubMed, Scopus, Web of Science, and the World Health Organization's compendium of protocols and guidelines pertaining to gestational diabetes. This strategy involved the identification and extraction of pertinent studies, followed by the elimination of duplicate entries.

Subsequently, a two-tiered screening process was conducted by two independent researchers to ascertain the relevance of the retrieved records. In the initial phase, the title and abstract of each record were scrutinized and those deemed incompatible with the study's objectives were excluded. In the subsequent phase, the full texts of the remaining records were thoroughly evaluated against the study's specific inclusion and exclusion criteria. Those satisfying these criteria were subsequently incorporated into the qualitative synthesis. (PRISMA protocol) [20].

### 2.2 Search strategy

A COVID-19 OR SARS-CoV-2 OR “Corona virus” OR COVID.
B Gestational OR Prenatal OR Pregnancy OR Antenatal OR Pregnancy-Induced.
C Diabetes OR “Diabetes Mellitus” OR insulin OR "insulin resistance" OR "Gestational Diabetes Mellitus".
D A AND B AND C.

### 2.3 Eligibility criteria

The inclusion criteria for this study were original randomized controlled trials (RCTs), cohort studies, and case-control studies that addressed changes to gestational diabetes mellitus (GDM) protocols, diagnostic processes, and treatments during the COVID-19 pandemic, from the start of the pandemic in December 2019 until January 1, 2022.

The period from December 2019 to January 2022 is significant as it marks the duration from the onset of the COVID-19 pandemic to the point where widespread vaccination efforts led to a reduction and containment of its spread.

Therefore, the exclusion criteria are as follows.

1) Non-original studies, including letters, reviews, commentaries, opinions, guidelines, or any studies with no original data.
2) Pure laboratory or animal studies not conducted on humans.
3) Duplicated results in databases.
4) Ongoing projects (e.g., articles discussing the protocol of a future study).
5) Abstracts or conference abstracts, or no available in full text.
6) Studies not conducted on gestational diabetes.
7) Studies not related to the COVID-19 Pandemic.
8) Non-English language studies.

### 2.4 Selection process of studies

After determining the search strategy for each database, all articles obtained from different databases were entered into EndNote X8 software. First, all duplicate and overlapping studies in different databases were removed. Then, the names of authors, institutions and journals of all studies were removed. In the next step, the title and abstract of the studies were examined and the studies not related to the subject were excluded. Finally, the full text of the remaining articles were carefully examined by the inclusion and exclusion criteria, and irrelevant studies were excluded. Qualitative evaluation of the selected articles could then begin. The quality of the selected articles was evaluated using the Newcastle-Ottawa Scale (NOS) [21], a tool designed for assessing non-randomized studies, including case-control and cohort research. The NOS employs a 'star system' to judge studies across three broad categories: the selection of the study groups, the comparability of the groups, and the ascertainment of the exposure or outcome for case-control or cohort studies, respectively. The objective of this scale is to create an instrument that offers a straightforward and convenient method for the quality assessment of non-randomized studies within systematic reviews. According to the NOS, scores range from 0 (minimum) to 9 (maximum), with articles achieving 6 or more stars considered to be low-risk and of high quality, while those scoring below 6 are deemed high-risk and of lower quality. The Jadad scale, a 5-point tool for assessing the quality of randomized trials, indicates that a score of three or more signifies superior quality. It is widely utilized for evaluating the quality of randomized controlled trials (RCTs). The scale comprises two questions each for randomization and blinding, and one question dedicated to the evaluation of the reporting of withdrawals and dropouts [22]. Any disagreements among the researchers were resolved by consulting a third researcher [21]. (See supplementary File 1).

### 2.5 Data extraction

Data from a previously prepared checklist was used which included the items’ first author, type of evidence/study, country, age, BMI, maternal outcome, fetal/neonatal outcome, screening test and results.

## 3 Results

**Fig. 1:**
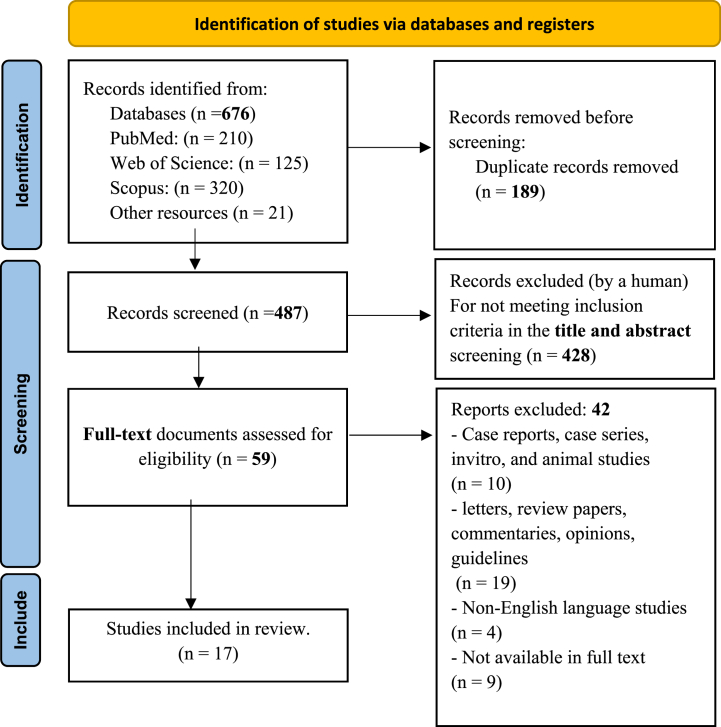
The PRISMA flowchart (2020).

In total, 676 publications were screened, 189 were duplicated and 487 were screened for title and abstract relevancy. 59 publications required full‐text review and of them, 17 studies fulfilled the inclusion criteria, as illustrated in Fig. 1.

### 3.1 Study characteristics

Seventeen studies were analyzed in the review. All the studies had acceptable scores in the risk of bias assessment (NOS). Eight studies were conducted on the population of pregnant women and the screening methods for diabetes during the COVID-19 pandemic were investigated (Table 1), and 9 studies examined different methods of gestational diabetes management (Table 2).

**Table 1:** Details of the changes of screening, diagnosis, and outcomes of GDM.

ID	First author	Type of Evidence/study	Country/Year	Participants			Maternal Outcome	Fetal/Neonatal Outcome	Screening/diagnosis test	Results
				N	Age (y)	BMI kg/m2				
1	van Gemert, T. E. (24)	Observational	Australia	16552	20-44 y	N/A	N/A	N/A	FBG	A third of women would go undiagnosed with GDM
2	d’Emden et al. (25)	Descriptive comparative	Australia	26242	N/A	N/A	- pregnancy - hypertension - Preterm labor - Primary Cesarean section	- LGA - Hyper insulinemia - adiposity	FBG A receiver operator characteristic (ROC (assessment	An effective method for determining glucose tolerance during pregnancy
3	Nachtergaele et al. (26)	Observational	France	4245	30.25 ± 5.32	24.36 ± 4.48	Preeclampsia Cesarean section	LGA Shoulder dystocia Neonatal hypoglycemia Preterm delivery	FBG	The initial FBG can eliminate the need for over 80 % of OGTTs.
4	McIntyre et al. (27)	Descriptive	Five HAPO study centers*	5974	29.4 ± 5.4	27.0 (24.2–31.0)	Pregnancy-related hypertension	Preterm LGA Hyperinsulinemia Neonatal hypoglycemia Neonatal adiposity	Alternative guideline for GDM screening (75 g of OGTTs and HbA1c)	All modified protocols implemented after COVID-19 have reduced the frequency of GDM.
5	Molina-Vega et al. (28)	Cohort	Spain 2019–2020	492	33.3 ± 5.6	28.9 ± 5.9	Hospitalization Obstetric trauma Hypertensive disorders of pregnancy	Preterm birth Neonatal hypoglycemia Phototherapy	Alternative guideline for GDM screening with two-stage diagnostic approach	There was no significant decrease in the prevalence of GDM.
6	van-de-l’Isle, Y. (29)	Retrospective cohort	UK	831 GDM = 8.5	N/A	N/A	N/A	Macrosomia Stillbirth	Alternative guideline for GDM according to RCOG recommended **stopping** the 2-h OGTT and having a two-step testing approach. First, women with risk factors for GDM are tested with HbA1c and RPG at booking. RPG ≥11.1 mmol/l is diagnostic of type 2 diabetes, and HbA1c value of 41–47 mmol/l is considered indicative of ‘pre-diabetes’.	The RCOG failed to identify 57 % of women and cannot be generally recommended
7	Meek et al. (30)	Retrospective cohort	UK	18923	31.7 ± 4.9	24.8 ± 5.0 29.4 ± 7.5	Caesarean section	LGA Neonatal hypoglycemia NICU admission	Alternative guideline for GDM screening (HbA1c, random plasma glucose, fasting plasma glucose, and plasma glucose and OGTT)	Fasting plasma glucose with a threshold of ≥5.2 mmol/L at 28 weeks proved to be the best predictor of gestational diabetes.
8	Kasuga, Y. (14)	Retrospective	Japan 2019	264	37 (23–51)	21.4	N/A	N/A	**Diagnosis with** IADPSG** **vs** Japanese GDM diagnostic strategy	The incidence of positive 1-h and 2-h glucose levels in the COVID-19-NFT (no further testing) group was significantly higher than in the COVID-19-GDM group (p < 0.01). In the Japanese GDM diagnostic strategy, which modifies criteria from other countries, fasting plasma glucose (FPG) and HbA1c are emphasized as crucial for diagnosing GDM.

**LGA**: large-for gestational age; **RCOG**: The Royal College of Obstetricians and Gynaecologists; **RPG**: random plasma glucose.

**Table 2:** Details of GDM management changes during COVID-19 pandemic.

ID	First author	Type of Evidence/study	Country/Year	Participants				Maternal Outcome	Fetal/Neonatal Outcome	Management	Results
				N	Mother Age (y)	Gestational age (w)	weight/BMI				
1	Wilk. et al. (9)	Retrospective	Poland	155	32.86 ± 4.25 year	23.7 ± 7.4 weeks	Pre-pregnancy 26.1 ± 5.2	No adverse outcomes	Adverse Outcomes were not significantly different.	Telemedicine	The assessment of diabetic care did not change significantly, but glycemia, self-monitoring, and the length of GDM training were significantly altered during the COVID-19 pandemic.
2	Dodesini et al. (31)	Retrospective	Italy 2020	14	35 ± 5	**--**	29.1 ± 5.6	N/A	N/A	Telehealth (to assess blood glucose trends) and CGM	The use of telehealth to monitor blood glucose trends, combined with continuous glucose monitoring (CGM), may have contributed to the relatively low number of pregnant women with pre-gestational diabetes who tested positive for COVID-19.
3	El Moazen et al. (36)	Clinical trial	Austria2020	27	32.6 (±5.6)	**-**	N/A	N/A	N/A	Telemedicine (tele monitoring)	Telemedicine is recommended as a therapeutic support for patients with GDM.
4	Albert et al. (33)	Descriptive	Spain	20	**-**	N/A	N/A	6 women had Caesarean section delivery, Preeclampsia	Macrosomia, Hypoglycemia, Jaundice, - Preterm birth, IUGR and Premature rupture of membranes	mobile phone application with AI (artificial intelligence) (the Sinedie app)	A mobile phone application automatically classifies and analyzes data such as ketonuria, dietary transgressions, and blood glucose values, and provides recommendations for adjustments to diet or insulin treatment.
5	Varnfield et al. (34)	Clinical trial	Australia/2017–2018	55	**-**	26.9	25.4	N/A	N/A	Use of mobile application (Mother platform) to manage GDM	It was useful for recording blood glucose levels, facilitated better support from the healthcare team, and was generally helpful in managing gestational diabetes.
6	Zhu, S. (16)	Retrospective	Australia	237	31.15 ± 5.69	**--**	25.10 ± 6.98	No difference was found.	There was no statistically significant difference between these 4 groups with respect to birth weight (p = 0.95).	**Standard management (**Diet, Metformin (MF), Insulin (MF + Insulin)	The most significant predictors of transitioning from diet to medication were the 2nd trimester fasting blood glucose (FBG) with an odds ratio of 3.58 and age with an odds ratio of 1.06.
7	Ghesquière et al. (35)	Retrospective	France	229	33.6 ± 4.9	**-**	28.6 ± 6.2	N/A	N/A	**Standard management** (Postprandial blood sugar, Use of insulin therapy)	-Diabetes control declined during COVID-19. -Postprandial blood sugar was significantly less well controlled in 2020 compared to 2019. -The use of insulin therapy was significantly higher in 2020 compared to 2019.
8	McIntyre et al. (27)	cohort	Multinational (USA, China, Australia, …)	5974	29.4 ± 5.4	GA: 24–32 weeks	BMI: 27.0 (24.2–31.0)	Pregnancy-related hypertension (48.4 %), Preterm Birth (79.5 %), Primary Cesarean section (83.8 %)	- Large-for-gestational age (79.8 %), - Neonatal hyper insulinemia (78.9 %), - Neonatal hypoglycemia (78.6 %) - Neonatal adiposity (74.7 %)	The modifications in GDM **diagnosis**	All modified pathways implemented post-COVID-19 reduced the frequency of GDM.
9	Chisini et al. (37)	Retrospective ecologic	Brazil	5564	–	N/A	N/A	Reduction in prenatal cares, diabetes and medical consultations performed in Primary Health Care	N/A	Integrating the care model (diabetes treatment, diabetic foot examination & determination of glucose in urine)	The number of diabetes treatment procedures significantly decreased

### 3.2 Gestational diabetes screening

Four studies were descriptive [8,[14], [15], [16]], and four were retrospective cohort studies [13,[23], [24], [25]]. 73,259 pregnant women of reproductive age participated in these studies (See Table 1).

Three studies utilized FBG as a screening test, yielding varying results. The results of them were different. One study suggested that using an FBG of 4.6 mmol/l or lower would result in missing almost a third (29 %) of women who would be diagnosed with GDM [15].

Results from another study indicated that the first FBG can prevent more than 80 % of OGTT with 49 % sensitivity for FBG. OGTT is performed only in women at risk (72 % sensitivity) [26]. The results of the third study showed FBG can be used effectively to define glucose tolerance in pregnancy during COVID-19 [27].

Four studies use the alternative guidelines for GDM screening, including plasma glucose 1 h and 2 h after 75 g of OGTTs [[23], [24], [28]] or a two-stage diagnostic approach (50 g OGTT & 100 g of OGT [25]. The results of these studies indicate plasma glucose 1 h and 2 h after 75 g of OGTTs protocols after COVID-19 reduced GDM frequency, therefore could not be recommended for general use. The use of a two-stage diagnostic approach has no significant reduction in GDM prevalence.

Remaining study compared guidelines in the diagnosis of diabetes during the COVID-19 pandemic [13]. Diagnosis using the International Association of Diabetes in Pregnancy Study Group (IADPSG) criteria was compared with the Japanese GDM diagnostic strategy. In the Japanese GDM diagnostic strategy, which is a modification of criteria from other countries, FPG (fasting plasma glucose) and HbA1c are listed as important for diagnosing GDM. According to the results for the diagnosis of COVID-19-GDM, a random cut-off of glucose levels (RPG ≥9.0 mmol/l (162 mg/dl)) may be inappropriate for Japanese GDM. The incidence of 1-h and 2-h glucose level positives in the COVID-19-NFT (no further testing) group were significantly higher than those in the COVID-19-GDM group (p < 0.01). The results of one study which used the modifications in GDM diagnosis and treatment indicated all post-COVID-19 modified pathways reduced GDM frequency [13].

### 3.3 Gestational diabetes management

Most of the studies that investigated diabetes monitoring in pregnancy were descriptive (n = 7, 63.63 %) [8,[29], [30], [31], [32], [33], [34]] (See Table 2). Two were cohort studies [13] and the other two were clinical trials [33,35]. The studies were conducted with pregnant women who had gestational diabetes. In total, there were 13,289 participants in the studies, with different gestational ages and BMI, which were not suitable for meta-analysis due to heterogeneity.

Telemedicine has served as a pivotal method for enabling patients to consult with their diabetic specialists, representing a notable shift from prior practices not widely adopted [8]. This approach is part of a broader, dynamic evolution of healthcare adjustments aimed at enhancing patient care. These adjustments include the use of telehealth and telephone consultations, designed with the dual objectives of improving pregnancy outcomes and safeguarding the well-being of pregnant women and healthcare staff [23]. In addition, the application of telehealth for monitoring blood glucose trends underscores its importance. The adoption of specific technologies, particularly Continuous Glucose Monitoring (CGM), alongside the implementation of telehealth protocols, has been instrumental in achieving a significantly low rate of COVID-19 cases among pregnant women with pre-gestational diabetes, particularly noted in Bergamo, Italy [30].

The mobile phone application was used in two studies to manage GDM [30,36], one of them based on artificial intelligence [30]. These apps automatically make adjustment recommendations regarding diet or insulin treatment rather than monitoring. They were useful in recording women's blood glucose levels (BGL), they facilitated better support of the health care team, and were generally helpful in managing gestational diabetes in pregnant women.

Two of the studies were based on standard treatment for diabetes management, including a healthy lifestyle (diet and exercise interventions) and drugs (Metformin and Insulin). Results indicated that diabetes control was lower during the COVID-19 epidemic and Insulin therapy was significantly higher in 2020 compared to 2019 [31,32]. A study used a mobile phone application with AI (artificial intelligence) that automatically classifies and analyses the data (ketonuria, diet transgressions, and blood glucose values), making adjustment recommendations regarding the diet or insulin treatment. The strongest predictor of moving from diet to medication was the 2nd trimester FBG (odds ratio of 3.58 and age of 1.06) [15].

An integrated care model was used in one research study that included diabetes treatment, diabetic foot examination, and determination of glucose in urine. The results of this study showed a reduction in medical appointments, which fell by 36 % and the number of diabetes treatment procedures fell significantly [34].

Maternal and fetal/neonatal adverse outcomes of GDM reported in some studies remained unchanged during the pandemic.

## 4 Discussion

The aim of the present study was to systematically review studies related to the changes of screening, diagnosis, outcomes and management of gestational diabetes management during the COVID-19.

GDM is a common problem in pregnancy. In relation to the COVID-19 pandemic, it might be prudent to attempt to reduce the number of women coming to a health service environment to try to limit the number of interpersonal contacts, in an attempt to minimize the risk of viral transmission. However, the implementation of such strategies must be judiciously evaluated considering the potential adverse consequences arising from undiagnosed and potentially inadequately managed GDM.

The salient outcomes derived from this comprehensive literature review encompass the following: (a) During the COVID-19 pandemic, a notable decrease was observed in the utilization of OGTTs, paralleled by a surge in the application of HbA1c tests, employed for the identification and clinical diagnosis of Gestational Diabetes Mellitus. (b) The predominant fraction of physicians employed diverse telemedicine methodologies for the remote surveillance and longitudinal management of patients afflicted with GDM.

Several studies have leveraged Fasting Blood Glucose (FBG) as a preliminary diagnostic tool [26], albeit with varying results. The updated guidelines for diagnosing GDM during the COVID-19 pandemic have the potential to reduce pregnant women's exposure to the virus. Nonetheless, they may overlook approximately one-third of all GDM cases. It is conceivable that women presenting with low fasting glucose levels yet displaying diagnostic levels at one and/or 2 h postprandial, may belong to a lesser risk stratum for unfavorable pregnancy outcomes. However, the evidence supporting this hypothesis is presently limited. A separate study indicated that an initial FBG measurement could obviate the need for more than 80 % of Oral Glucose Tolerance Tests (OGTTs), given the 49 % sensitivity of FBG. OGTTs are typically administered solely to women deemed at risk, revealing a sensitivity of 72 % [26]. Moreover, FBG proves to be a robust tool for delineating glucose tolerance during gestation amid the prevalence of the COVID-19 disease [27]. Zhu et al. (2021) described elevated FBG as a significant predictor for the need for medical management and proposed its potential for improving individualized treatment. They also indicated that HbA1c and FBG were ineffective screening tests for GDM during the COVID-19 pandemic. Therefore, OGTT should be prioritized clinically for high-risk patients [15]. Despite OGTT traditionally being perceived as the definitive diagnostic tool, the majority of contemporary guidelines have now acknowledged the necessity of diminishing OGTT utilization during antenatal and postpartum periods to minimize potential exposure during prenatal consultations. On a global scale, prompted by the escalating apprehension surrounding the COVID-19 pandemic, a number of professional organizations have disseminated advisories and directives advocating for alterations in the diagnostic procedures and therapeutic strategies for GDM throughout the duration of the COVID-19 pandemic [23]. A review conducted by Nouhjah et al. (2020) outlined that temporary changes in clinical guidelines have been updated to align with social distancing and reduce COVID-19 exposure risk. Despite ongoing controversies in gestational diabetes management, addressing the pandemic has facilitated a global consensus on these matters [37].

The revised guidelines for the diagnostic pathway of pregnant women being tested for GDM during the temporary COVID-19 pandemic will certainly lower the number of women who are potentially exposed [14]. However, it is estimated that about a third of all cases will be missing. There is limited data available on whether women with low fasting levels but diagnostic one- or 2-h levels are in a lower risk group for adverse pregnancy outcomes. However, this remains a possibility.

Throughout the duration of the COVID-19 pandemic, enforced domestic seclusion has amplified the circumstances surrounding pregnant women diagnosed with GDM, culminating in less auspicious pregnancy outcomes. These encompass diminished Apgar scores, elevated incidence rates of cesarean deliveries, and an increased prevalence of macrosomia and nuchal cord occurrence [38].

In consideration of the multitude of women displaying reluctance to undertake OGTT due to the extended exposure duration within clinical or hospital environments, and the subsequent elevated risk of contracting COVID-19, both the United Kingdom and Canada have proposed circumventing the use of OGTT [39,40]. The United Kingdom has promoted an approach reliant on risk-factor based testing, categorizing women with HbA1c levels at or above 5.7 %, FPG at or exceeding 100 mg/dL (5.6 mmol/L), or a random measurement at or surpassing 162 mg/dL (9 mmol/L) as being "at risk" [40]. Contrarily, Canadian guidelines propose a slightly divergent metric, utilizing HbA1c ≥ 5.7 % and/or a random measure at or exceeding 200 mg/dL (11.1 mmol/L) GDM screening during the pandemic [41].

In contrast to the UK and Canadian position, Australian policy does not fully eliminate the need for OGTT. Their recommendations denote an FPG between 85 and 90 mg/dL (4.7–5.0 mmol/L) as an indicator for a full OGTT, while an FPG above 92 mg/dL (5.1 mmol/L) is considered confirmatory for GDM. Conversely, an FPG lower than 85 mg/dL (4.7 mmol/L) is interpreted as non-GDM [39]. In circumstances such as these, domestic glucose surveillance employing capillary glucose monitoring or continuous interstitial fluid glucose monitoring may be executed for a duration spanning one to two weeks, or potentially for the remaining gestational period [42].

Given the increased susceptibility of pregnant women to severe complications of COVID-19, which include preterm birth, preeclampsia, cesarean section, and perinatal death, clinical services should be tailored to maximize patient safety. This can be achieved by maintaining social distancing, enforcing appropriate utilization of personal protective equipment, and employing telemedicine strategies [43]. For those presenting with manifest diabetes, it is crucial to conduct tests for kidney and thyroid function, Glycated Hemoglobin (HbA1c), and Protein/Creatinine Ratio (PCR) in urine at the initial consultation. In-person appointments should be minimized, and when essential, they should be scheduled during the 11–14, 28–32, and 34–36 week gestation periods, coinciding with obligatory tests and ultrasonography sessions [44]. In light of extended waiting times at health or medical facilities due to resource constraints during the pandemic, the performance of a 2-h oral glucose tolerance test for gestational diabetes screening was deemed inadvisable. As an alternative, it was recommended to utilize HbA1c and random plasma glucose tests [45].

In the study conducted by Xie et al., the authors assessed the efficacy of telemedicine interventions in managing gestational diabetes through a comprehensive meta-analysis encompassing 32 randomized controlled trials. The results indicated that telemedicine, in comparison to conventional care, significantly attenuates glycemic levels in individuals with gestational diabetes and concurrently mitigates the incidence of complications among mothers, neonates, and fetuses [9].

Based on studies in pregnant women with gestational diabetes, telemedicine services, when compared to usual care, were associated with lower rates of premature births, premature rupture of fetal membranes, neonatal asphyxia, and polyhydramnios [9]. While in terms of quality of life, average gestational age at delivery, average birth weight, LGA, newborn's first and fifth minute Apgar score and hyperbilirubinemia, there was no difference in the use of telemedicine compared to usual care [46,47].

Female individuals afflicted with diabetes have articulated several advantages arising from the employment of mobile health (mHealth) technologies during gestation. The refinement of upcoming digital solutions through collaborative design processes, involving end-users, could potentially ameliorate the perceived constraints and augment the efficacy of mHealth technology [48].

There was disagreement about the effectiveness of telemedicine compared to usual care for some parameters. While improvements in cesarean section rate, 2-h blood glucose, and fasting blood glucose were observed with the use of telemedicine, some studies reported no difference in these parameters compared to receiving usual care [9,46,47].

Additionally, the results of some studies showed telemedicine to be beneficial in improving HbA1c control, ketonuria, adherence to diet, blood glucose levels and insulin levels [32].

Three studies used telemedicine to assess blood glucose trends and telemonitoring [8,23,29]. The results of these studies indicate telemedicine may help pregnant women with diabetes as a therapeutic support for GDM patients.

The mobile app for managing GDM was utilized in two studies [30,36], with one involving artificial intelligence [30]. These apps provide automatic adjustment suggestions for diet or insulin treatment instead of just monitoring. They proved beneficial for tracking BGL (blood glucose levels), enhancing healthcare team support, and effectively managing gestational diabetes in pregnant mothers.

Standard treatments for diabetes management, such as a healthy lifestyle (diet and exercise interventions) and medications (Metformin and Insulin), were utilized in two of the studies. The findings showed that during the COVID-19 epidemic, the effectiveness of diabetes control decreased, and there was a substantial increase in the use of Insulin therapy in 2020 versus 2019 [31,32]. The strongest predictor of moving from diet to medication was the 2nd trimester FBG (odds ratio of 3.58 and Age of 1.06).

The research conducted by Banerjee et al. identified several impediments to telemedicine adoption, encompassing attitudinal, communicational, cultural, socio-economic, and legal factors [49]. Health systems could alleviate financial burdens by integrating telehealth services [50].

Rasekaba et al. also found in 2018 that telemedicine support for GDM care had no effect on service utilization and cost. Clinical outcomes in the telemedicine service group were similar to usual care and posed no risk to the quality of clinical care. This intervention is possible with lower insulin dose titration and the participants achieved optimal blood sugar control sooner [9].

Applications and telemedicine can be beneficial, however, reducing care and limiting tests during the COVID-19 pandemic may lead to mothers missing check-ups and could result in complications. Therefore, it is preferable to maintain the same standards as pre-pandemic, while ensuring full adherence to guidelines. Other studies used an alternative order for GDM screening including plasma glucose 1-h and 2-h after 75 g OGTTs or a two-step diagnostic (50 g OGTT and 100 g OGT. Studies show that plasma glucose 1-h and 2 -hours after 75 g OGTT protocols after COVID-19 show a reduced frequency of GDM.

### 4.1 Strengths and limitations

This review is one of the first in the literature to address this critical issue, offering substantial benefits for pregnant women and public health, particularly in the context of potential future pandemics. Its primary strength is its pioneering nature, as it sheds light on a previously unexplored area, providing valuable insights that can guide clinical practice and inform policymaking. Additionally, the review's focus on the impact of the pandemic on the diagnosis, screening and management of GDM is exceptionally timely and relevant, given the ongoing changes in the pandemic and their implications for maternal and fetal health.

However, a notable limitation of this systematic review is the relatively few numbers of studies involved in the final analysis, which may affect the robustness and generalizability of the findings. Additionally, the heterogeneity of the included studies may pose challenges in drawing definitive conclusions. Furthermore, the review's reliance on available evidence at a specific point in time may limit its ability to capture the full spectrum of the impact of the COVID-19 pandemic on GDM management and diagnosis. Despite these limitations, the review's emphasis on telemedical approaches and digital interventions in managing GDM during the pandemic represents a forward-looking approach that aligns with the evolving landscape of healthcare delivery in the context of public health crises. Finally, our systematic review on changes in screening, diagnosis, outcome and managing GDM during COVID-19 fills a crucial gap in the literature but also highlights the necessity for ongoing research. The findings offer insights into evidence-based GDM strategies during such pandemics and contribute to protecting maternal and child health in public health crises.

## 5 Conclusion

The COVID-19 pandemic necessitated alterations in the screening, diagnosis, and management protocols for GDM. Monitoring women diagnosed with GDM during the pandemic is crucial to understand their pregnancy outcomes and evaluate their prospective diabetes risk. Key adaptations include.

∗ Application of a 'single test procedure' for GDM diagnosis: This method allows for the GDM evaluation to be completed in a single visit, involving a 75-g oral glucose load and the estimation of plasma glucose from a blood sample taken 2 h post-ingestion.
  - The glucose load can be consumed at home in certain circumstances, with the expectant woman visiting the healthcare facility 2 h later for a single sample for plasma glucose estimation.
  - If visiting a laboratory is unfeasible, a plasma glucose standardized glucometer can be used. This streamlined procedure facilitates swift GDM diagnosis and reduces the infection risk for antenatal women.
∗ Integration of telemedicine and digital technology: Effective management of GDM during pandemics can leverage technology to mitigate challenges.
  - Even without sophisticated software and database systems, healthcare providers, including diabetologists and obstetricians/gynecologists, can use electronic medical records or telephone communications for monitoring and patient follow-ups.

These strategies underscore the importance of innovative approaches to healthcare delivery in managing GDM amidst pandemic challenges.

## Funding

Student Research Committee, Deputy for Research and Technology, Kermanshah University of Medical Sciences (IR) (50001433), research project No. IR.KUMS.REC.1402.213.

## Ethics approval and consent to participate

The project was found to be in accordance to the ethical principles and the national norms and standards for conducting Medical Research in Iran.

## Consent for publication

Not applicable.

## Data availability statement

The data that support the findings of this study are available on request from the corresponding author.

## CRediT authorship contribution statement

**Kowsar Qaderi:** Writing – original draft. **Ahmadreza Shamsabadi:** Formal analysis, Data curation. **Arezoo Haseli:** Methodology. **Sajjad Ghane Ezabadi:** Validation. **Leila Asadi:** Conceptualization. **Younes Jesmani:** Conceptualization. **Mehri Kalhor:** Methodology. **Bita Jamali:** Methodology. **Mehrnaz Kajbafvala:** Formal analysis. **Rasa khodavirdilou:** Validation, Methodology. **Aida Mohammadi:** Data curation, Conceptualization. **Dara Rasoal:** Writing – review & editing, Writing – original draft, Methodology.

## Declaration of generative AI and AI-assisted technologies in the writing process

During the revision of this work the author(s) used [ChatGPT 4] in order to [improve readability and language]. After using this tool/service, the author(s) reviewed and edited the content as needed and take(s) full responsibility for the content of the publication.

## Declaration of competing interest

The authors declare that they have no known competing financial interests or personal relationships that could have appeared to influence the work reported in this paper.
