# Supplementary material for: Changes in screening, diagnosis, management, and outcomes of gestational diabetes during the COVID-19 pandemic: A systematic review
Source: Heliyon. 2024 May 25;10(11):e31943. doi: 10.1016/j.heliyon.2024.e31943 (PMC11154622; doi:10.1016/j.heliyon.2024.e31943)
Supplement: Multimedia component 1 [file mmc1.docx]

Supplementary File 1: Newcastle-Ottawa Quality Assessment Form for **Cohort Studies**

| **Total**  **(9*)** | **Outcome** | | | **Comparability*** | **Selection** | | | | **Study ID** |
| --- | --- | --- | --- | --- | --- | --- | --- | --- | --- |
|  |  |  |  |  |  | | | |  |
|  | **follow-up long enough for outcomes to occur (*)** | **Adequacy of follow up (*)** | **Assessment of outcome (*)** | **(**)** | **Demonstration that outcome of interest was not present at start of study (*)** | **Ascertainment of exposure (*)** | **Selection of non-exposed cohort (*)** | **Representativeness of exposed cohort (*)** |  |
| **7** | ***** | ***** | ***** | **-** | **-** | ***** | ***** | ***** | Chisini, LA. (35) |
| **8** | **-** | ***** | ***** | ***** | ***** | ***** | ***** | ***** | McIntyre, HD. (25) |
| **8** | ***** | ***** | ***** | ***** | **-** | ***** | ***** | ***** | Ghesquière, L. (33) |
| **8** | ***** | ***** | ***** | ***** | ***** | ***** | ***** | ***** | Kasuga, Y. (14) |
| **8** | ***** | ***** | ***** | ***** | ***** | ***** | ***** | ***** | Molina-Vega, M. (26) |
| **9** | ***** | ***** | ***** | ****** | ***** | ***** | ***** | ***** | van-de-l’Isle, Y. (27) |
| **8** | ***** | ***** | ***** | ***** | ***** | ***** | ***** | ***** | Meek, C.L (28) |
| **8** | ***** | ***** | ***** | ***** | ***** | ***** | ***** | ***** | Wilk, M. (9) |
| **7** | ***** | ***** | ***** | ***** | ***** | ***** | **-** | ***** | Dodesini, A. R. (29) |
| **8** | ***** | ***** | ***** | ***** | ***** | ***** | ***** | ***** | Zhu, S. (16) |
| **7** | * | * | * | * | * | * | - | * | Albert, L. (31) |
| **8** | * | * | * | * | * | * | * | * | d’Emden, M. (23) |
| **8** | * | * | * | - | * | * | * | * | van Gemert, T. E. (22) |
| **9** | * | * | * | * | ** | * | * | * | Nachtergaele, (24) |

Title: Newcastle-Ottawa Quality Assessment Form **for cross sectional studies**

**Title: Quality assessment of RCT studies via Jadad scale†**

| **Study ID** | **random sequence generation** | **allocation concealment** | **Double-blindness** | **Dropout and reasons** | **Total score** |
| --- | --- | --- | --- | --- | --- |
| El Moazen, G. (34) | 1 | 1 | 1 | 1 | 4 |
| Varnfield, M. (30) | 1 | 1 | 1 | 1 | 4 |
